# 2D-nanomaterials for AKI treatment

**DOI:** 10.3389/fbioe.2023.1159989

**Published:** 2023-03-09

**Authors:** Qiaohui Chen, Xiaoyuan Wang, Chao Yuan, Yayun Nan, Qiong Huang, Kelong Ai

**Affiliations:** ^1^ Department of Pharmacy, Xiangya Hospital, Central South University, Changsha, Hunan, China; ^2^ Xiangya School of Pharmaceutical Sciences, Central South University, Changsha, Hunan, China; ^3^ Hunan Provincial Key Laboratory of Cardiovascular Research, Xiangya School of Pharmaceutical Sciences, Central South University, Changsha, China; ^4^ Geriatric Medical Center, People’s Hospital of Ningxia Hui Autonomous Region, Yinchuan, Ningxia, China; ^5^ National Clinical Research Center for Geriatric Disorders, Xiangya Hospital, Central South University, Changsha, Hunan, China

**Keywords:** two-dimension, nanomaterials, targeted therapy, acute kidney injury, antioxidant therapy

## Abstract

Acute kidney injury has always been considered a sword of Damocles over hospitalized patients and has received increasing attention due to its high morbidity, elevated mortality, and poor prognosis. Hence, AKI has a serious detrimental impact not only on the patients, but also on the whole society and the associated health insurance systems. Redox imbalance caused by bursts of reactive oxygen species at the renal tubules is the key cause of the structural and functional impairment of the kidney during AKI. Unfortunately, the failure of conventional antioxidant drugs complicates the clinical management of AKI, which is limited to mild supportive therapies. Nanotechnology-mediated antioxidant therapies represent a promising strategy for AKI management. In recent years, two-dimensional (2D) nanomaterials, a new subtype of nanomaterials with ultrathin layer structure, have shown significant advantages in AKI therapy owing to their ultrathin structure, large specific surface area, and unique kidney targeting. Herein, we review recent progress in the development of various 2D nanomaterials for AKI therapy, including DNA origami, germanene, and MXene; moreover, we discuss current opportunities and future challenges in the field, aiming to provide new insights and theoretical support for the development of novel 2D nanomaterials for AKI treatment.

## Introduction

Acute kidney injury, characterized by a sudden loss of renal function and rapid increases in serum creatinine (Cre) and urea nitrogen levels (BUN), is a common and critical clinical condition involving multiple etiologies (Kellum et al., 2021). AKI has long been a significant factor influencing ICU patient prognosis, with a prevalence of 30%–60% and a high correlation with fatal events (Pickkers et al., 2021). Especially in the context of the COVID-19 pandemic, AKI often complicates the hospitalization of patients diagnosed with the virus and may lead to increased disease severity, prolonged hospitalization, and poor prognosis (Ronco et al., 2020). Unfortunately, current clinical support therapies such as dialysis, rehydration, and renal transplantation do not fundamentally stop the progression of the disease, but merely relieve symptoms and wait for the self-repair of kidneys. (Ostermann et al., 2020). Therefore, focusing on the common pathological mechanisms of AKI and providing targeted drug therapies is an effective strategy to stop the progression of the disease and promote renal repair (Chen et al., 2023). Emerging evidence has revealed that the key mechanism of AKI is an uncontrolled burst of toxic reactive oxygen species (ROS) in kidneys, which initiates a chain reaction involving cell apoptosis, necrosis, and excessive inflammation (Wu et al., 2019; Zhao et al., 2021a; Chen et al., 2023). As the second energy-consuming organ of the body, renal tubules with a high density of mitochondria become the main site of ROS production and the target of attack during ischemia or hypoxia (Tian and Liang, 2021; Li et al., 2022). ROS storms induce severe oxidative stress, causing irreversible damage to DNA, proteins, and other biomolecules, and ultimately leading to tubular cell dysfunction, i.e., a sudden decline in kidney function (Weng et al., 2021; Zhao et al., 2022a; Huang et al., 2023a; Wang et al., 2023a; Su et al., 2023). Therefore, kidney-targeted scavenging of excess ROS offers a new potential solution for AKI treatment (Wang et al., 2021a; Wang et al., 2022). However, typical antioxidants such as *N*-acetylcysteine (NAC) have produced highly mixed results in clinical studies due to rapid excretion, poor bioavailability and low ROS scavenging efficacy (Su et al., 2017; Zhang et al., 2021a). Accordingly, biosafe nanomedicines are highly desirable for the treatment of AKI, because of their high kidney-targeting ability and strong antioxidant (Zhou et al., 2020).

Since the beginning of the 21st century, the successful exfoliation of graphene has triggered the explosive growth of two-dimensional (2D) nanomaterials, which have two dimensions outside the nanoscale and only one dimension with one thickness or a few atomic layers (Choi et al., 2022). Most atoms in 2D nanomaterials are directly exposed on their surfaces, endowing these materials with the largest specific surface area and extraordinary surface activity (Ouyang et al., 2022). Notably, 2D nanomaterials can maintain their atomic thickness while retaining their large longitudinal dimensions. Compared to zero-dimensional (0D) or larger-size nanomaterials, 2D nanomaterials have many unique features, such as lamellar structures, excellent optical/ultrasonic/magnetic responses, and high thermal conversion efficiency, which endow them with considerable potential and several advantages in the biomedical field (Hu et al., 2019; Zhao et al., 2022b). Therefore, they can act as antioxidants with maximum exposure for efficient ROS scavenging and possess abundant anchoring sites, which enable them to serve as drug carriers through diverse functional modifications while improving the drug loading rates (Zhang et al., 2020). Most importantly, the ultrathin and flexible structure of 2D nanomaterials endows them with unique preferential renal excretion properties, allowing them to coil and fold longitudinally across the glomerular filtration barrier (Jiang et al., 2020). Therefore, 2D nanomaterials with high surface activity and natural kidney targeting are highly promising candidates for antioxidant therapy of AKI.

**FIGURE 1:**
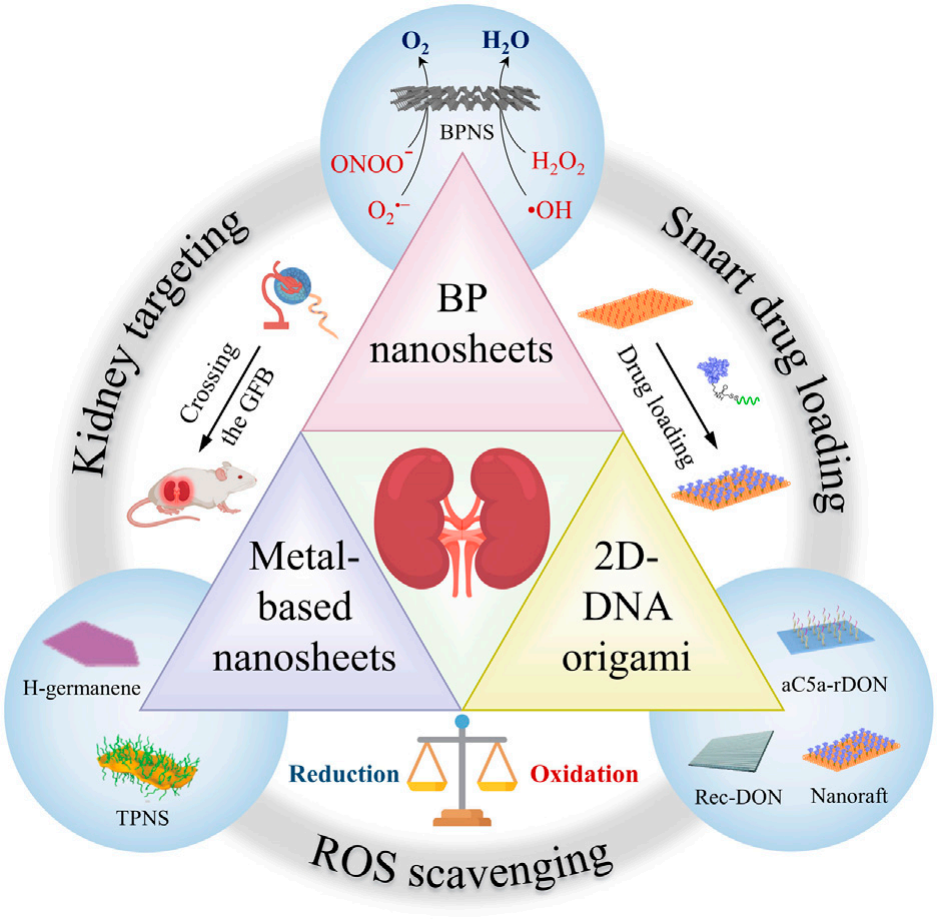
Schematic illustration of 2D nanomaterials for AKI treatment. In recent years, several emerging 2D nanomaterials have demonstrated excellent therapeutic effects in AKI due to their excellent kidney targeting, smart drug loading and ROS scavenging capabilities, including 2D-DNA origami, metal-based nanosheets and BP nanosheets.

Over the past few years, the extensive development of 2D nanomaterials has provided fertile ground for the exploration of high-performance AKI therapeutic nanomedicines. However, to the best of our knowledge, no reviews of 2D nanomaterials for AKI treatment have been published to date. To fill this gap, in this minireview we summarize recent progresses on 2D nanomaterials for AKI antioxidant therapy, including 2D DNA origami, metal-based and black phosphorus (BP) nanosheets (Figure 1; Table 1). We focus on the structural design, biodistribution characteristics, reactivity, and biosafety of these 2D nanomaterials. Finally, we discuss the challenges and prospects of the application of 2D nanomaterials in related fields, with the aim of triggering further innovative researches to advance the development of AKI therapeutics.

**TABLE 1:** 2D-nanomaterials in AKI treatment.

Category	Nanomaterials	Size	Thickness	Cargo	Therapeutic mechanism	Animal model
2D-DNA origami	Rec-DON Jiang et al. (2018)	90 × 60 nm	—	—	Antioxidation	Rhabdomyolysis induced AKI
	aC5a-rDON Chen et al. (2021)	90 × 60 nm	—	aC5a	Antioxidative and Anti-inflammation	Ischemia reperfusion induced AKI
	Nanoraft Li et al. (2021b)	—	4 nm	IL-33	Antioxidation and Anti-inflammation	Ischemia reperfusion induced AKI
Metal-based nanosheets	H-germanene Chen et al. (2022)	100–400 nm	<1 nm	—	Antioxidation	Rhabdomyolysis induced AKI
	TPNS Zhao et al. (2021c)	∼200 nm	—	—	Antioxidation and Anti-inflammation	Rhabdomyolysis induced AKI
Black phosphorus nanosheets	BPNSs Hou et al. (2020)	225.8 ± 4.0 nm	3.8–4.5 nm	—	Antioxidation	Rhabdomyolysis induced AKI

### 2D-DNA origami

Over the past three decades, significant advances have been made in structural DNA nanotechnology, enabling the fabrication of highly programmable DNA origami with unprecedented accuracy and complexity, making them ideal precision nanomaterials (Wang et al., 2023b; Parsons et al., 2023). In addition, DNA is particularly advantageous in biomedical applications, owing to its intrinsic biocompatibility and biodegradability as the transporter of genetic information in living organisms (Li et al., 2021a; Zhang et al., 2021b; Lucas et al., 2022). DNA origami structures have been widely investigated and applied in various biomedical fields, including tissue engineering, immune engineering, drug delivery, diagnosis, and biosensing (Tian et al., 2022; Zhang et al., 2022). Importantly, the nucleophilic of the DNA bases could effectively neutralize toxic ROS, allowing DNA to be employed as an exogenous ROS scavenger for antioxidant therapy of AKI (Chen et al., 2023).

Recently, Jiang et al. reported that rectangular 2D-DNA origami exhibited preferential renal accumulation properties as well as low immunogenicity and minimal cytotoxicity (Jiang et al., 2018). The authors fabricated tightly folded DNA origami with three distinct morphologies (Rec-DON, Tub-DON, and Tri-DON), along with the corresponding partially folded structures through a single-step annealing procedure. After radiolabeling, all three intact DNA origami structures exhibited preferential renal accumulation in both healthy and AKI mice, while the partially folded structures exhibited increased liver sequestration like M13 ssDNA (long single-stranded DNA). The similar biodistribution patterns of partially folded DNA strictures and M13 ssDNA indicated the rapid recognition and clearance of circulating foreign antigens by the endothelial reticular system. In contrast, the dense structure, high degree of folding, and negative surface charge of the intact DNA origami efficiently avoided foreign DNA-triggered immunosurveillance or enzyme/protein interactions, as well as the subsequent liver sequestration. More significantly, the ultra-thin and soft morphology of the 2D-DNA origami structures enabled them to pass through the glomerulus filtration barrier upon coiling and folding, which ultimately led to their accumulation at the target, i.e., the renal tubules. Moreover, all types of DNA origami effectively neutralized various types of ROS (•OH, O_2_^•−^, and H_2_O_2_) through the oxidation of DNA bases. Rec-DNA, with the highest kidney uptake, effectively restored the redox balance (superoxide dismutase levels), and renal function (Cre, BUN) in AKI mice at an effective dose of only 1/420 of NAC (one of the commonly used clinical antioxidants).

Furthermore, the ultra-high specific surface area and precision of DNA origami technology also enable the application of 2D-DNA origami as smart drug delivery devices with optimized loading rates and targeting efficiency (Jiang et al., 2019). Tailored 2D-DNA nanorobots for kidney-targeted drug delivery were easily obtained by rationally arranging capture strands as anchoring sites on the surface of nanosheets (Thai et al., 2020; Zhao et al., 2021b). For example, Chen et al. reported a rectangular DNA origami (aC5a-rDNAs) loaded with nucleic acid aptamers of complement component (C5a) for the sequential therapy of AKI (Chen et al., 2021). In agreement with previous studies, the rDNAs exhibited preferential renal accumulation *in vivo*, and their persistence time in AKI mice could exceed 12 h post-injection due to their decreased renal function, compared to only 3 h in healthy mice. Such high drug retention provided a longer therapeutic window and was a prerequisite for achieving sequential treatment. During AKI, excessive ROS can also cause excessive inflammatory responses by activating the complement system along with direct oxidative injury. Therefore, the aC5a-rDNAs enabled stage-sensitive sequential therapy based on the direct link between oxidative stress and complement activation. Accordingly, aC5a-rDNAs could scavenge excess ROS during the first stage of AKI (0–4 h) *via* the oxidation of DNA bases, displayed as a significantly reduced malondialdehyde (MDA) level in renal tissue. When the AKI advanced to the second stage (4–8 h), the nucleic acid aptamers on the surface of aC5a-rDNAs specifically bound C5a and competitively inhibited the activation of the complement system, which in turn decreased the expression of myeloperoxidase, tumor necrosis factor-α, interleukin-6, and interleukin-1 expression in renal tissues. As a result, aC5a-rDNAs possessing renal targeting and bifunctionality properties exhibited a superior therapeutic effect compared with bare rDNAs and free aC5a, with significantly recovered and improved renal function indicators (BUN, Cre) and renal tissue morphology.

Cytokine interleukin-33 (IL-33) is a powerful immunomodulator that plays a crucial role in suppressing inflammation and promoting tissue repair (Dagher et al., 2020; Faas et al., 2021). However, its non-specific activation on immune cells outside the focus and short circulating half-life represent major obstacles for its application in AKI therapy. Recently, Li et al. constructed a DNA nanoraft-based cytokine delivery platform by precisely arranging IL-33 arrays on rectangular DNA origami, aiming to achieve preferentially kidney delivery of IL-33 with accurate dosage and sustained drug release (Li et al., 2021b). Compared to free IL-33, nanorafts showed a predominant renal distribution with the fluorescent signal that lasted for more than 48 h in the kidney, indicating that nanorafts can significantly increase the specific renal distribution and retention time of IL-33 in the kidney. During the process, the fluorescence intensity and co-localization of nanorafts with IL-33 in the kidney decreased with time, reflecting the slow and continuous release of IL-33 from the surface of nanorafts. As an important immunomodulator, IL-33 could subsequently induce rapid expansion of type 2 innate lymphoid cells (ILC2s) and regulatory T cells (Tregs) by binding to their specific surface receptors ST2. As a result, the treatment with nanorafts led to a significant increase in the percentage and absolute number of renal ILC2s and Tregs, accompanied by an upregulated expression of IL-4 and IL-13, which play important roles in repolarization of macrophages from the pro-inflammatory M1 subtype to the anti-inflammatory M2 subtype. Furthermore, the renal function indices (Cre, BUN) and tubular injury score of nanoraft-treated mice (two times injection at 2 and 48 h after surgery) were better than those of the free-IL-33-treated group (five consecutive days of injection after surgery). Taking these results together, the authors offered a structurally well-defined delivery platform for controlled cytokine immunotherapy, achieving a higher therapeutic efficiency with less treatment intensity in AKI treatment.

### Metal-based nanosheets

Germanene, group-IV graphene-like 2D buckled nanosheets, has recently received considerable attention as one of the newest members of 2D nanomaterials (Feng et al., 2020; Rohaizad et al., 2021). Germanium is a frequently employed trace element in the human body, with many crucial biological functions, including erythropoiesis as well as antibacterial, anticancer, antiviral, and immunoregulation activities, suggesting its high biocompatibility and potential medical value (Ge et al., 2021). However, the biomedical applications of germanene remain largely unexplored. Recently, Chen et al. reported the design of 2D germanene nanosheets and their antioxidant application for AKI therapy. Hydrogen-terminated germanene (H-germanene) was synthesized through Ca layer deintercalation from the precursor Zintl-phase CaGe_2_ crystals followed by delamination and ultrasonic treatment (Chen et al., 2022). The hydrogenation strategy tuned the band gap of germanene, allowing H-germanene to be employed as an electron donor and thus serve as an antioxidant. As a result, ultrathin H-germanene (<1 nm) exhibited superior broad-spectrum ROS scavenging ability (H_2_O_2_, O_2_^•−^, •OH) and ultra-high reaction efficiency compared to typical antioxidant nanoparticles such as CeO_2_, Au, TiO_2_, and MnO_2_. After intravenous injection, H-germanene rapidly accumulated in the kidneys of AKI mice and maintained high renal concentrations for the first 3 h. The passive targeting further amplified the ROS scavenging effect of H-germanene, and the corresponding treatment significantly reduced the levels of the DNA oxidation product 8-OhdG and the lipid peroxidation product MDA in kidney tissues of AKI mice. The restoration of renal function indices (Cre, BUN) in AKI mice further demonstrated the excellent efficacy of H-germanene nanosheets. In addition, the injection of high-concentration H-germanene showed no effect on body weight, hematological parameters, or liver and kidney function indices in mice, which demonstrated the biosafety of H-germanene and its potential for further clinical applications.

MXenes are a class of two-dimensional inorganic compounds that consist of atomically thin layers of transition metal carbides, nitrides, or carbonitrides (Mostafavi and Iravani, 2022). As an emerging branch of the 2D material family, MXenes have drawn substantial interest in biomedical applications due to their planar structure and unique physicochemical properties such as surface hydrophilicity, optical/magnetic/thermal properties, and abundant surface functional groups (Lin et al., 2018; Soleymaniha et al., 2019). More importantly, 2D Ti_3_C_2_ MXenes display strong chemical reactivity toward ROS, which makes them effective antioxidants for the treatment of AKI (Wang et al., 2021b; Hou et al., 2022). Recently, Zhao et al. reported ultrathin Ti_3_C_2_-PVP nanosheets (TPNs) and explored their therapeutic potential in AKI (Zhao et al., 2021c). The modification of (polyvinylpyrrolidone) PVP on the surface of TPNs improved the colloidal stability and dispersion of the nanosheets under physiological conditions, because of the steric hindrance of the macromolecular chains. Through adsorption and reduction at [Ti_3_C] and Ti top sites, TPNs additionally displayed broad-spectrum (H_2_O_2_, O_2_^•−^, •OH) and powerful ROS scavenging ability. In particular, they exhibited intrinsic enzyme/H_2_O_2_-responsive triggered biodegradability, with significant size reduction and subsequent degradation into Ti^2+^, Ti^3+^, and Ti^4+^ oxides (TiO_x_ species), ensuring their excellent biodegradability and biosafety. Owing to their planar structure, TPNs showed preferential renal accumulation at 5 min post-injection, peaking at 12 h. After treatment with TPNs, the renal function indices (BUN, Cre) and histopathological sections of AKI mice demonstrated their excellent therapeutic effects. Furthermore, transcriptomic and WB analyses showed that the TPNs served as a powerful antioxidant platform to scavenge extra ROS and then attenuate oxidative stress-induced inflammatory responses in AKI by inhibiting the NF-κB signaling pathway, illustrating their high clinical translational potential in AKI and other ROS-related diseases.

### Black phosphorus nanosheets

Layered BP, a new member of the 2D nanomaterial family, has received a warm reception from scientists due to its unique physicochemical characteristics, controllable size, and excellent surface activity (Hu et al., 2020; Sui et al., 2021). In the field of optoelectronics, layered BPs undergo severe degradation and gradually lose their original properties when exposed to air or aqueous solutions, hindering their practical applications (Thurakkal and Zhang, 2020). However, such drawbacks can be turned into a great advantage in the biomedical field. The responsive degradation property makes BP a very competitive candidate compared to other 2D nanomaterials, because it minimizes the long-term toxicity and poor excretion issues when exposed to the physiological environment. Recently, Hou et al. used liquid-phase exfoliation to prepare black phosphorus nanosheets (BPNSs) with 7–9 individual BP layers for AKI antioxidant treatment (Hou et al., 2020). Each P atom in the individual BP layers is covalently bonded with three neighboring P atoms by sp^3^ hybridization, forming a puckered honeycomb structure, and the adjacent layers are weakly stacked through van der Waals interactions. Due to their stacked layer structure (enabling rapid electron transfer) and elemental state (facilitating a rapid oxidative reaction to generate P–O bonds), the BPNSs exhibit a great capacity for ROS consumption. In AKI mice, BPNSs demonstrated excellent renal targeting ability thanks to their high planar/thickness topology and the suppressed renal clearance; after 12 h of intravenous administration, the renal distribution of BPNSs was up to 80%. The preferential accumulation of BPNS at the focal site maximized their antioxidant effect in AKI mice, and their nephroprotective effect was superior to that of two clinical antioxidant drugs (amifostine and NAC) at the same dose. Additionally, HE staining results and TUNEL fluorescence images demonstrated less tissue damage and apoptosis in BPNS group. Notably, after fulfilling their protective role, BPNSs can be readily degraded to biocompatible P_x_O_y_ ions after reacting with ROS, guaranteeing their excellent biosafety.

## Conclusion and prospects

Despite the rapid advances in clinical care using modern biomedical technologies, dealing with serious and complex diseases such as AKI is still a major challenge. To combat these dangerous diseases, it is essential to develop creative therapeutic strategies. Nanomedicine is an innovative technology for designing and synthesizing various materials with fascinating physicochemical properties at the sub-micron level and exploiting them in the biomedical field (Liu et al., 2022a; Zhu et al., 2022; Huang et al., 2023b; Sun et al., 2023). Multiple medical fields are already benefiting from the advantages offered by nanotechnology, including disease diagnosis and surveillance, cancer therapy, and regenerative medicine, etc (van der Meel et al., 2019; Yang et al., 2019; Xu et al., 2020; Liu et al., 2022b). Encouragingly, outstanding progress in the development of novel antioxidant nanodrugs for AKI has been achieved in the past decades through the concerted efforts of many research groups (Chen et al., 2023). Considering the failure of conventional small-molecule antioxidant drugs, specific renal targeting ability, efficient and broad-spectrum ROS scavenging activity, and excellent biocompatibility have become the core principles of the design of therapeutic nanodrugs for AKI therapy. In these studies, 2D nanomaterials with their flexible and ultra-thin lamellar structure and well-exposed antioxidant active sites have demonstrated their unique appeal in AKI treatment, highlighting the “structure-function” correlation. Despite these encouraging developments, 2D nanomaterials still have a long way to go before further clinical translation.

Firstly, it has been founded that the size, thickness, composition, and surface properties of 2D nanomaterials are closely related to their biological behavior after entering the body (Fan et al., 2022). However, apart from the synthesis of DNA origami following a well-defined and controllable procedure, it is still challenging to achieve the controlled synthesis of 2D nanomaterials with adjustable size, uniform thickness, and stable dispersion. For example, liquid-phase exfoliation is currently the most common synthesis method of 2D material; however, despite its easy application, it is challenging to use this approach for the production of high-quality 2D materials, owing to aggregation, inhomogeneous morphology, random surface distribution, and size restriction issues (Alam et al., 2021). Secondly, although substantial evidence demonstrates the preferential kidney accumulation properties of 2D nanomaterials, their exact pharmacokinetic behavior, including the exact retention time in the focus and the actual clearance mechanism, remains unclear (Zheng et al., 2021). On the one hand, the large surface area of 2D nanomaterials increases their ability of the materials to interact with human tissues, which may complicate their metabolism (Fan et al., 2022). On the other hand, the novelty of some 2D nanomaterials also implies that limited information is available on the physiological characteristics of their interactions with living tissues. As a result, beyond the incomplete understanding gained so far, a more systematic assessment of the *in vivo* fate of 2D nanomaterials is needed. Thirdly, as an intelligent drug-delivery platform for targeting kidney tissues, the controlled drug release of 2D nanomaterials deserves further exploration and improvement. The development of DNA origami structures has enabled precise and quantitative control of drug loading and oxidative decomposition of DNA molecules by ROS to be achieved upon drug release; however, it is difficult to completely avoid premature drug leakage outside the focus and stable drug release at the focus. Future studies may aim to utilize the optical, thermal, magnetic, or microenvironmental response of 2D materials to control the drug concentration at the target site and achieve the optimal therapeutic impact.

Despite the many challenges involved, the special physiochemical and kidney targeting properties of 2D nanomaterials continue to attract interest for their applications in kidney disease treatment. In addition to the excellent work discussed above, 2D nanomaterials require a broader range of material innovations in the field of AKI therapy. We expect that additional 2D nanomaterials with great biocompatibility, such as carbon-based 2D materials, silicate clays, and transition metal disulfides, will be investigated for the treatment of oxidative stress-related illnesses. Furthermore, innovative combinations of 2D nanomaterials with different dimensions provide new development opportunities for these systems. For example, Xu et al. designed a novel 2D DNA origami equipped with a microRNA-responsive one-dimensional nanoantenna, which enables the smart early diagnosis of AKI through PA imaging (Xu et al., 2022). Further 2D smart nanostructures with responsive, multifunctional, and programmable properties are expected to be developed in the future, which will lead to novel concepts and approaches for the treatment of kidney diseases such as AKI.
