# Corrigendum: 2D-nanomaterials for AKI treatment

**DOI:** 10.3389/fbioe.2023.1199818

**Published:** 2023-04-18

**Authors:** Qiaohui Chen, Xiaoyuan Wang, Chao Yuan, Yayun Nan, Qiong Huang, Kelong Ai

**Affiliations:** ^1^ Department of Pharmacy, Xiangya Hospital, Central South University, Changsha, Hunan, China; ^2^ Xiangya School of Pharmaceutical Sciences, Central South University, Changsha, Hunan, China; ^3^ Hunan Provincial Key Laboratory of Cardiovascular Research, Xiangya School of Pharmaceutical Sciences, Central South University, Changsha, China; ^4^ Geriatric Medical Center, People’s Hospital of Ningxia Hui Autonomous Region, Yinchuan, Ningxia, China; ^5^ National Clinical Research Center for Geriatric Disorders, Xiangya Hospital, Central South University, Changsha, Hunan, China

**Keywords:** two-dimension, nanomaterials, targeted therapy, acute kidney injury, antioxidant therapy

In the published article, there was an error in the Funding statement. The funding statement for the Key Program of Ningxia Hui Autonomous Region Natural Science Foundation of China was displayed as “No. 2022JJ21059”. The correct Funding statement appears below.

“The Key Program of Ningxia Hui Autonomous Region Natural Science Foundation of China (No. 2022AAC02058).”

The authors apologize for this error and state that this does not change the scientific conclusions of the article in any way. The original article has been updated.

